# Opioid prescriptions among the World Trade Center Health Program population

**DOI:** 10.1186/s12913-023-10233-z

**Published:** 2023-11-30

**Authors:** Ruiling Liu, Geoffrey M. Calvert, Kristi R. Anderson, Helen Malcolm, Lauren Cimineri, Hannah Dupont, Marisol Martinez

**Affiliations:** World Trade Center Health Program, National Institute for Occupational Safety and Health (NIOSH), Centers for Disease Control and Prevention (CDC), Atlanta, GA 30329 USA

**Keywords:** Outpatient opioid prescription, Prescription indicator, World Trade Center Health Program, Quality improvement

## Abstract

**Background:**

The World Trade Center Health Program (Program) provides limited health care to those directly affected by the 9/11 terrorist attacks. Because of physical/mental trauma arising from the 9/11 attacks, Program members might be at high risk of opioid use. To prevent prescription opioid overuse, in 2018 the Program implemented various measures to improve opioid prescribing and expand access to non-opioid pain management among Program members. However, the characteristics of opioid prescriptions dispensed among this population has never been described.

**Methods:**

Administrative and claims data from 07/01/2011 to 09/30/2022 were used to describe opioid prescriptions dispensed during 2013–2021.

**Results:**

From 2013–2021, 108,285 members were Program-enrolled for ≥ 10 months, 4,053 (3.7%) had 22,938 outpatient opioid prescriptions, of which, 62.1% were for cancer-related pain, 11.1% for hospice/end of life care, 4.8% for surgery pain, and 9.8% for acute/chronic pain. Among members with Program-paid diagnostic/treatment claims (*n* = 70,721), the proportion with opioid prescriptions for cancer/hospice/end of life care increased from 0.5% in 2013 to 1.6% in 2018 (*p* = 0.010), then decreased to 1.1% in 2021 (*p* = 0.070), and the proportion for non-cancer surgery/acute/chronic pain decreased from 0.6% in 2013 to 0.23% in 2021 (*p* = 0.0005). Among members prescribed opioids without cancer/hospice/sickle cell disease, the proportion who started with long-acting opioids or had opioid prescriptions from ≥ 4 prescribers were below 6.5% annually; the proportion receiving a high-dose (≥ 90 morphine milligram equivalents per day [MED]), or with concurrent opioids and benzodiazepines use, or who started opioids with MED ≥ 50 or with long duration (≥ 7 days’ supply) were above 10% annually, but decreased since 2017.

**Conclusions:**

Prevalence of outpatient opioid prescriptions paid by the Program was very low and prescriptions were primarily dispensed for cancer/hospice/end of life care. Although Program efforts to improve opioid prescribing coincided with improvements in outcomes, ongoing surveillance is needed.

## Background

The opioid overdose epidemic is one of the most pressing public health challenges in the United States. Opioid-involved overdose deaths increased from 21,089 in 2010 to 80,411 in 2021, and overdose deaths involving natural and semisynthetic opioids (e.g., morphine, oxycodone, hydrocodone), a category inclusive of prescription opioids, rose from 3,479 in 2001 to a peak of 14,495 in 2017, with 13,618 in 2021 [1]. Risk factors associated with prescription opioid misuse include younger age (< 40 years old), male sex, current or history of other substance use, and some mental health conditions such as depression, anxiety [2], and post-traumatic stress disorder (PTSD) [2, 3]. Receiving opioids from multiple (four or more) prescribers and pharmacies is also associated with opioid overdose [4].

To improve communication between clinicians and patients about the risks and benefits of opioid therapy for chronic pain, the Centers for Disease Control and Prevention (CDC) published *CDC Guideline for Prescribing Opioids for Chronic Pain* in 2016 which included 12 recommendations for prescribing opioids for chronic pain among outpatients without cancer, palliative care, or end-of-life care [5]. In 2022, CDC released a new guideline (*Clinical Practice Guideline for Prescribing Opioids for Pain*), expanding and updating the 2016 CDC Guideline to provide recommendations to help ensure persons have equitable access to safe and effective pain management that improves function and quality of life while illuminating and reducing risks associated with prescription opioids for outpatients without cancer, palliative care, or end-of-life care [6].

The World Trade Center (WTC) Health Program (Program) was established in 2011 by the James Zadroga 9/11 Health and Compensation Act of 2010 (Zadroga Act) and reauthorized in 2015 (42 United States Code [U.S.C.] §§ 300 mm – 300 mm-61) to provide health care to those directly affected by the 9/11 attacks. The Program is a limited health benefit program, which covers monitoring and treatment for specified health conditions resulting from exposures related to the 9/11 terrorist attacks in New York, the Pentagon, and in Shanksville, Pennsylvania. To become a Program member and receive Program health benefits, one must meet certain exposure, latency, and symptom-onset requirements for listed conditions.

Individuals potentially eligible to enroll in the Program include emergency responders and other workers or volunteers who were directly involved in the rescue, recovery, clean-up, and related support activities following the attack (collectively referred to as “responders”), and those who were present in the New York City disaster area on 9/11 or its aftermath because of their work, residence, or attendance at school or day care (referred to as “survivors”). It is estimated that potentially eligible individuals include more than 17,000 responders from the Fire Department of New York City (FDNY), more than 80,000 non-FDNY general responders, and more than 360,000 survivors [7]. As on July 31, 2022, a total of 118,849 members (83,970 responders and 34,979 survivors) were enrolled in the Program, including essentially all FDNY responders, approximately 80% of non-FDNY general responders, and about 10% of survivors [8]. Of the potentially eligible survivors, 300,000 are thought to be passersby and persons in transit on 9/11 [7], and few of these persons are thought to be enrolled given the difficulty of proving that they were exposed. Once enrolled, members stay with the Program for their lifetime unless they are disenrolled due to Program error, member submission of inaccurate eligibility information or member request to withdraw from the Program. As of the end of 2022, only 12 members have been disenrolled from the Program.

Program members are a unique population because of their environmental exposures and physical and mental trauma arising from the terrorist attacks. As such, this population has an increased risk for certain covered health conditions, including, but not limited to, aerodigestive, musculoskeletal, mental health, and cancer conditions [9, 10]. A member’s health condition must be certified for coverage by the Program for treatment to be covered.

To improve opioid prescribing and expand access to non-opioid pain management among members, the Program implemented various measures beginning in September 2018, such as: I) first opioid prescription cannot exceed a 7-day supply and implemented a point-of-sale limit on opioid prescriptions with > 90 morphine milligram equivalents (MMEs) per day; II) added coverage for non-opioid pain management (e.g., physical therapy, acupuncture and neurostimulation), and removed coverage of opioids with high misuse or overdose potential, specifically methadone and all oral fentanyl products; III) semi-annually reviewed controlled substance prescription data to identify potential fraud, waste, and abuse; and IV) periodically identified members with opioids prescriptions that were considered potentially high-risk: those who receive opioid prescriptions from two or more prescribers or two or more pharmacies, and members with average daily opioid utilization > 90 MMEs, and/or received concurrent treatment with opioids and benzodiazepines. For those members who were prescribed opioids identified as potentially high-risk, the Program follows up with their providers to review their medical records and help to ensure their opioid prescriptions are clinically indicated.

To align with recommendations in the 2016 CDC Guideline, the Pharmacy Quality Alliance [11], a national quality organization dedicated to improving medication safety, adherence and appropriate use, developed eight opioid measures based on claims data to evaluate health plan performance on opioid prescribing. Four PQA measures evaluate use of opioids at high dosage, from multiple prescribers and pharmacies, and concurrently with benzodiazepines, which are associated with an increased risk of potentially fatal opioid overdose [12]. Three initial opioid prescribing PQA measures evaluate new prescriptions at high dosage, for long duration, or for long-acting or extended release opioids, which should be reserved for severe, continuous pain and are associated with a higher risk of overdose compared to immediate-release opioids [6, 13]. One PQA measure evaluates annual drug monitoring among individuals prescribed long-term opioid therapy. All these PQA measures exclude individuals with a cancer diagnosis, sickle cell disease, or hospice care.

The intent of our study was to use the Program’s administrative (members’ enrollment and medical certifications) and medical and pharmaceutical claims data to characterize opioid prescriptions dispensed among Program members, and to estimate the PQA measures to explore areas that may benefit from improvements in opioid prescribing for members without a cancer diagnosis, sickle cell disease, or hospice care. The Program’s experience may help provide insights to other health programs in managing opioid prescribing.

## Methods

### Data source

Administrative and claims data routinely collected by the Program were used for this analysis on Program members’ opioid prescriptions during 2013–2021. Member enrollment data include demographic information (age, sex, and race/ethnicity). Certification data include members’ Program-certified health conditions and the date of certification, which is generally when the member satisfies 9/11 exposure criteria and a Program-affiliated physician attests that those exposures were substantially likely to have been a significant factor in aggravating, contributing to, or causing the health condition [14]. Medical claims data include information collected via the Centers for Medicare & Medicaid Service CMS-1500 Claim Form for professional claims or the UB-04 Claim Form for institutional claims. Each claim line includes one Current Procedural Terminology (CPT) code for medical services and procedures [15], service date, and up to 12 International Classification of Disease, Clinical Modification (ICD-CM) codes. Some claim lines also have a revenue code indicating the department or place in which the procedure was performed. Pharmaceutical claims data include Program-paid outpatient prescriptions’ National Drug Codes (NDCs) [16], prescription written date, dispense date, quantity dispensed, and days of supply. Data collected through 09/30/2022 were used to identify claims for medical services provided through 12/31/2021 to account for the time lag in claim submission and processing.

### Data analysis

Members enrolled for at least 10 months with the Program during 2013–2021 were included. This 10-month requirement was adopted because the PQA manual recommends individuals to be enrolled in a health care program for at least 10 months in a measurement year to be included in estimates of measures related to opioid prescription. This 10-month requirement also accounts for the time lag between Program enrollment and getting certified for a WTC condition (certification is required to receive Program covered condition-related treatment). For this population, opioid prescription prevalence from 2013 through 2021 was estimated for all members and different subgroups defined by age, sex, race/ethnicity, member type (responders or survivors), Program-certified conditions, with or without diagnostic/treatment-related claims, and with or without cancer (excluding non-melanoma skin cancer, which was not analyzed in this paper), hospice care, surgery pain, acute pain, or chronic pain.

ICD-9-CM codes 140–239 (except 173), or ICD-10-CM codes C00-D50 (except C44) were used to identify members with WTC certifications for cancer and to identify medical claims with a cancer diagnosis. Members with surgical procedures, acute pain, chronic pain or sickle cell disease were identified as those with any claims that had a surgery CPT code, or an ICD-10-CM code for corresponding conditions listed by Mikosz, et al. [17] or a corresponding ICD-9-CM code derived from a crosswalk published by CMS [18]. Members with hospice care were defined as those with any medical claims that included a CPT code G0182, G9473-G9479, Q5003-Q5008, Q5010, S9126, T2042-T2046, 99377–99378, or a revenue code of 0115, 0125, 0135, 0145, 0155, 0235, 0650–0652, 0655–0659, which were provided by PQA.

Opioid NDCs and their oral MME conventional factors provided by CDC [19] and PQA were used to identify opioid outpatient prescriptions from pharmaceutical claims paid by the Program, and to calculate MMEs per day (MED). Opioids of cough and cold formulations, including certain elixirs/syrup and combination products containing antitussives, decongestants, antihistamines, and expectorants, were excluded from this analysis. Injectable formulations, sublingual sufentanil, and all buprenorphine products were also excluded. MED was calculated using the following equation [19]:

$$MED=\;\left(Strength\;per\;Unit\;of\;Drug\;\times Quantity\;Dispensed/Days'\;Supply\right)\times MME\;conversion\;factor$$

For each outpatient opioid prescription, it was assumed that use began on the dispense date and continued daily until the last day of supply. If a member had any day covered by more than one opioid prescription based on this assumption, those days were counted only once when calculating the length of opioid use, and the MED was the sum of MEDs of all prescriptions on that date.

**Fig. 1:**
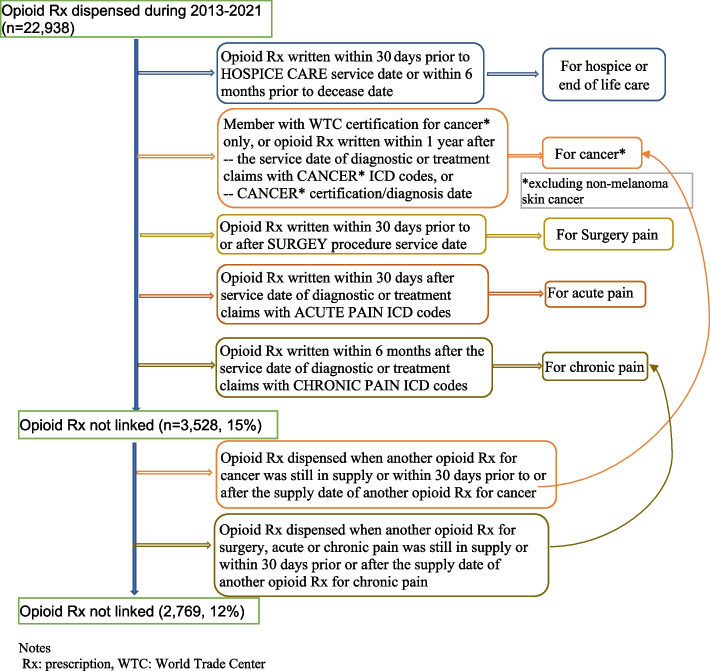
Opioid prescription indicator algorithms

To understand the purpose of an opioid prescription, the written/dispense date was linked to the member’s medical claims and WTC condition certifications, and an indication was assigned to each prescription in the hierarchy of hospice/end of life care, cancer, surgery pain, acute pain, or chronic pain. The algorithms are shown in Fig. 1, adapted from the algorithms developed by Mikosz et al., which were reviewed by an Opioid Prescribing Estimates Workgroup comprised of experts from a variety of specialties, physicians and non-physician practitioners and patient representatives [17].

Simple linear regression analyses were conducted to test the trend of the proportion of members with diagnostic or treatment-related claims who had opioid prescriptions for different purposes, with year as the independent variable and proportion as the dependent variable. A *p* value of 0.05 was used to determine whether a trend was statistically significant or not.

### PQA measures

PQA measures on opioid prescriptions by year from 2013 to 2021 were estimated following the manual provided by PQA through licensing. Descriptions of the measures and methods are shown in Table 1. These measures exclude members with any medical claims for hospice care, cancer, or sickle cell disease during the measurement period. Note that sickle cell disease is not a condition that is covered by the Program, and only a few members had claims with this condition listed in the non-primary diagnosis fields.

**Table 1:** Pharmacy Quality Alliance (PQA) opioid measure descriptions and estimate methods

Measure name	Description	Eligible Population (Denominator)	Numerator
Use of Opioids at High Dosage in Persons Without Cancer (OHD)	The percentage of individuals ≥ 18 years of age who received prescriptions for opioids with an average daily dosage of ≥ 90 morphine milligram equivalents (MME) over a period of ≥ 90 days	Individual ≥ 18 years old on the 1st day of year, who had ≥ 2 opioid prescriptions dispensed, ≥ 15 days' cumulative supply, and an opioid episode (from the first date of supply of the first opioid prescription dispensed to the last date of supply of the last opioid prescription dispensed in the measurement year) ≥ 90 days in the year, excluding those with any claims for hospice care, cancer, or sickle cell disease in the year	Individuals from the denominator who received opioid prescriptions with an average daily dosage of ≥ 90 MME over a period of ≥ 90 days
Use of Opioids from Multiple Providers in Persons Without Cancer (OMP)	The percentage of individuals ≥ 18 years of age who received prescriptions for opioids from ≥ 4 prescribers AND ≥ 4 pharmacies within ≤ 180 days		Individuals from the denominator who received opioid prescriptions from ≥ 4 unique prescribers AND ≥ 4 unique pharmacies within 180 days or through the end of the opioid episode, whichever is shorter
Use of Opioids at High Dosage and from Multiple Providers in Persons Without Cancer (OHDMP)	The percentage of individuals ≥ 18 years of age who received prescriptions for opioids with an average daily dosage of ≥ 90 MME AND who received prescriptions for opioids from ≥ 4 prescribers AND ≥ 4 pharmacies		Individuals from the denominator who received opioid prescriptions with an average daily dosage of ≥ 90 MME over a period of ≥ 90 days and who received opioid prescriptions from ≥ 4 unique prescribers AND ≥ 4 unique pharmacies within 180 days or through the end of the opioid episode, whichever is shorter
Concurrent Use of Opioids and Benzodiazepines (COB)	The percentage of individuals ≥ 18 years of age with concurrent use of prescription opioids and benzodiazepines	Individual ≥ 18 years old on the 1st day of year, who had ≥ 2 opioid prescription dispenses, ≥ 15 days' cumulative supply in the year, excluding those with any claims for hospice care, cancer, or sickle cell disease in the year	Individuals from the denominator who had ≥ 2 prescription claims with different dates of service for any benzodiazepines in the year, and had concurrent use of opioids and benzodiazepines for ≥ 30 cumulative days
Initial Opioid Prescribing at High Dosage (IOP-HD)	The percentage of individuals ≥ 18 years of age with ≥ 1 initial opioid prescriptions with an average daily MME of ≥ 50	Individual ≥ 18 years old on the 1st day of year, who had ≥ 1 opioid prescription dispensed in the year and no opioid medication during 90 days before the dispense date (initial opioid prescriptions), excluding those with any claims for hospice care, cancer, or sickle cell disease in the year or 90 days prior to 1st opioid dispense date	Individuals from the denominator with an average daily MME ≥ 50 for all opioid prescriptions dispensed within any opioid initiation period (i.e., initial opioid prescription dispense date + 6 days) in the year
Initial Opioid Prescribing for Long Duration (IOP-LD)	The percentage of individuals ≥ 18 years of age with ≥ 1 initial opioid prescriptions for > 7 cumulative days’ supply		Individuals from the denominator with > 7 cumulative days’ supply for all opioid prescriptions dispensed during any opioid initiation period (i.e., initial opioid prescription dispense date + 2 days) in the year
Initial Opioid Prescribing for Long-Acting or Extended-Release Opioids (IOP-LA)	The percentage of individuals ≥ 18 years of age with ≥ 1 initial opioid prescriptions for long-acting or extended-release (LA/ER) opioids		Individuals from the denominator with ≥ 1 LA/ER opioid prescription claim during any opioid initiation period (i.e., initial opioid prescription dispensed date + 6 days) in the year
Annual Monitoring for Persons on Long-Term Opioid Therapy (AMO)	The percentage of individuals ≥ 18 years of age who are prescribed long-term opioid therapy and have not received a drug test at least once during the measurement year	Individual ≥ 18 years old on the 1st day of year, who had ≥ 90 days’ cumulative supply of opioids during the measurement year, excluding those with any claims for hospice care, cancer, or sickle cell disease in the year or 90 days prior to 1st opioid dispense date	Individuals in the denominator who have not received a drug test during the measurement year

## Results

### Characteristics of WTC Health Program members who were prescribed opioids and opioid prescriptions during 2013–2021

A total of 108,285 members were enrolled for at least 10 months in the Program during 2013–2021 and included in the analysis. In this sample, 78.3% were males, 91.6% were 18–65 years old on 01/01/2013, 52.0% were white, 13.0% Hispanic and 8.9% black, and 73.5% were responders and 26.5% survivors (Table 2). As of 12/31/2021, 61.9% had at least one Program-certified health condition, 19.1% were certified for cancer, 9.9% for PTSD (no cancer), 0.7% for substance use disorder (SUD) (no PTSD or cancer), 4.9% for other mental health conditions, and 8.2% for obstructive sleep apnea (OSA). A total of 70,721 (65.3%) members had diagnostic- or treatment-related claims paid by the Program during 2013–2021, and 11,638 (10.8%) had claims with acute or chronic pain ICD-CM code(s). A total of 4,053 (3.7%) members had at least one outpatient opioid prescription paid by the Program during 2013–2021 (Table 2). The annual opioid prescription rate increased from 0.6% in 2013 to 1.1% in 2017, and then decreased to 0.6% in 2021 (data not shown). Males (compared to females), responders (compared to survivors), non-Hispanic white (compared to other race/ethnicity groups), members certified for cancer, PTSD, other mental health conditions, or OSA (compared to those with other or no Program certified conditions), and members with hospice care, surgery, acute or chronic pain claims (compared to those without those claims) had higher percentages of opioid prescriptions (Table 2).

**Table 2:** World Trade Center (WTC) Health Program member characteristics

	**Members enrolled for ≥ 10 months in 2013–2021 (a)**		**Members with outpatient opioid prescriptions dispensed in 2013–2021 (b)**	
**Categories**	**N**	**% (95% CI)**	**N**	**% (95%CI) (b/a)**
**All**	108,285	100.00	4,053	3.74 (3.62, 3.86)
**Sex**				
Female	23,434	21.64 (21.40, 21.89)	626	2.67 (2.46, 2.88)
Male	84,834	78.34 (78.10, 78.59)	3,427	4.04 (3.91, 4.17)
Unknown	17	0.02 (0.01, 0.02)		
**Age as of 01/01/2013**				
18–40 years old	15,599	14.41 (14.20, 14.61)	357	2.29 (2.05, 2.52)
41–65 years old	83,016	76.66 (76.41, 76.92)	3,377	4.07 (3.93, 4.20)
> 65 years old	9,094	8.40 (8.23, 8.56)	319	3.51 (3.13, 3.89)
Unknown	576	0.53 (0.49, 0.58)		
**Race/Ethnicity, combined**				
Hispanic	14,000	12.93 (12.73, 13.13)	505	3.61 (3.30, 3.92)
White	56,196	51.90 (51.60, 52.19)	2,722	4.84 (4.67, 5.02)
Black	9,607	8.87 (8.70, 9.04)	279	2.90 (2.57, 3.24)
Other^a^	4,557	4.21 (4.09, 4.33)	111	2.44 (1.99, 2.88)
Unknown	23,925	22.09 (21.85, 22.34)	436	1.82 (1.65, 1.99)
**Member type**				
Responder	79,594	73.50 (73.24, 73.77)	3,494	4.39 (4.25, 4.53)
Survivor	28,691	26.50 (26.23, 26.76)	559	1.95 (1.79, 2.11)
**Certified WTC conditions as of 12/31/2021**				
Cancer^b^	20,638	19.06 (18.83, 19.29)	2,469	11.96 (11.52, 12.41)
PTSD^c^ (no cancer^b^)	10,670	9.85 (9.68, 10.03)	574	5.38 (4.95, 5.81)
SUD^d^ (no PTSD/cancer^b^)	777	0.72 (0.67, 0.77)	17	2.19 (1.16, 3.22)
Other mental health condition(s)^e^ (no PTSD/SUD/cancer^b^)	5,252	4.85 (4.72, 4.98)	193	3.67 (3.17, 4.18)
OSA^f^ (no cancer or mental health conditions)	8,836	8.16 (8.00, 8.32)	433	4.90 (4.45, 5.35)
Other WTC condition only^g^	20,835	19.24 (19.01, 19.48)	358	1.72 (1.54, 1.89)
None	41,277	38.12 (37.83, 38.41)	9	0.02 (0.01, 0.04)
**With diagnosis or treatment claims**	70,721	65.31 (65.03, 65.59)	4,053	5.73 (5.56, 5.90)
**With hospice/end of life care**	305	0.28 (0.25, 0.31)	199	65.25 (59.90, 70.59)
**With WTC certification or medical claims for cancer**^**b**^	23,537	21.74 (21.49, 21.98)	2,708	11.51 (11.10, 11.91)
**With surgery procedures**	2,968	2.74 (2.64, 2.84)	1,393	46.93 (45.14, 48.73)
**With acute/chronic pain claims**^**h**^	11,638	10.75 (10.56, 10.93)	2,198	18.89 (18.18, 19.60)

^a^Other includes Asian (*n* = 3,100), American Indian or Alaskan Native (214), Native Hawaiian/Pacific Islander (*n* = 93), multi-racial (*n* = 775), other (*n* = 375)

^b^cancer, malignant, not including non-melanoma skin cancer

^c^PTSD: post-traumatic stress disorder

^d^SUD: substance use disorder, including alcohol use disorder, drug and other substance use disorder, including 31 members certified for opioid use disorder

^e^Other mental health conditions covered by the WTC Health Program, including adjustment disorders, anxiety, and depression

^f^OSA: obstructive sleep apnea

^g^Including acute/traumatic injuries, airway and digestive disorders, and musculoskeletal conditions that are associated with the 911 event

^h^Claims with any acute or chronic pain related ICD-9-CM or ICD-10-CM code listed

A total of 22,938 opioid prescriptions were dispensed to 4,053 members, equivalent to an average of five prescriptions dispensed per member receiving opioids (median = 1, range 1–271), or 21 opioid prescriptions dispensed per 100 Program members. About 25% (*n* = 5,797) of the prescriptions dispensed had 1–7 days’ supply, 23% (*n* = 5,205) had 8–29 days’ supply, 52% (*n* = 11,909) had 30 days’ supply, and only 0.1% (*n* = 27) had > 30 days’ supply (data not shown). About 66% (*n* = 15,088) of the prescriptions were dispensed on the same day when they were written, and another 24% (*n* = 5,598) were dispensed within one week (Table 3). Sixteen percent (*n* = 737) of members who were prescribed opioids had at least one high-dose prescription (≥ 90 MMEs/day), and those high-dose prescriptions accounted for 29.8% (*n* = 6,834) of the total opioid prescriptions. Three quarters (*n* = 17,282) of the prescriptions were short-acting opioids, and the rest were long-acting opioids. More than 20% (*n* = 1,118) of members receiving opioid had “new” opioid prescriptions dispensed after more than 90 days of no known opioid prescriptions. Most opioid prescriptions were for cancer-related pain (62%, *n* = 14,260); more than 10% (*n* = 2,555) were for hospice or end-of-life care, 5% (*n* = 1,098) for surgery pain, and 9% (2,256) for acute or chronic pain (Table 3).

**Table 3:** Features of opioid prescription claims paid by the WTC Health Program during 2013–2021

	**Members who received opioids**		**Opioid prescriptions dispensed**		**Number of opioid prescriptions dispensed per member who received opioid**^**g**^	
**Categories**	**N**	**%**	**N**	**%**	**Mean (95%CI)**	**Median (range)**
**All**	4,053	100.0	22,938	100.0	5.66 (5.46, 5.85)	1 (1, 271)
**Sex**						
Female	626	15.45	3,436	14.98	5.49 (4.62, 6.36)	2 (1, 104)
Male	3,427	84.55	19,502	85.02	5.69 (5.16, 6.22)	1 (1, 271)
**Age at opioid prescription dispense date**^**a**^						
18–40 years old	114	2.74	494	2.15	4.33 (2.19, 6.47)	1 (1, 89)
41–65 years old	3,256	78.33	18,531	80.79	5.69 (5.16, 6.22)	1 (1, 233)
> 65 years old	787	18.93	3,913	17.06	4.97 (4.28, 5.67)	2 (1, 114)
**Race/Ethnicity**^**b**^						
Hispanic	505	12.46	2,082	9.08	4.12 (3.26, 4.99)	1 (1, 92)
White	2,722	67.16	15,981	69.67	5.87 (5.27, 6.47)	1 (1, 271)
Black	279	6.88	1,220	5.32	4.37 (3.50, 5.24)	1 (1, 77)
Other	111	2.74	570	2.48	5.14 (3.10, 7.17)	2 (1, 66)
Unknown	436	10.76	3,085	13.45	7.08 (5.39, 8.77)	2 (1, 233)
**Member type**						
Responder	3,494	86.21	19,039	83.00	5.45 (4.96, 5.95)	1 (1, 271)
Survivor	559	13.79	3,899	17.00	6.94 (5.57, 8.31)	2 (1, 233)
**Time from prescription written to dispense**						
Same day	3,452	61.51	15,088	65.78	4.37 (4.02, 4.72)	1 (1, 210)
Within 7 days	1,539	27.42	5,598	24.40	3.64 (3.30, 3.97)	1 (1, 80)
More than 7 days	603	10.74	2,206	9.62	3.66 (3.10, 4.22)	1 (1, 93)
Unknown (missing written date)	18	0.32	46	0.20	2.56 (1.06, 4.05)	1 (1, 12)
**Single dispense with ≥ 90 MMEs/day**^**c**^						
Yes	737	15.83	6,834	29.79	9.27 (7.72, 10.83)	2 (1, 220)
No	3,918	84.17	16,104	70.21	4.11 (3.83, 4.39)	1 (1, 127)
**With short-acting opioid**	3,996	86.47	17,282	75.34	4.32 (4.01, 4.64)	1 (1, 163)
**With long-acting opioid**	625	13.53	5,656	24.66	9.05 (7.85, 10.25)	4 (1, 115)
**With long term opioid therapy**^**d**^						
Yes	708	16.66	16,199	70.62	22.88 (20.67, 25.09)	12 (1, 271)
No	3,542	83.34	6,739	29.38	1.90 (1.83, 1.97)	1 (1, 52)
**With new opioid prescription**^**e**^** (no opioid prescriptions in previous 90 days)**						
Yes	1,118	21.89	1,940	8.46	1.74 (1.66, 1.81)	1 (1, 13)
No	3,989	78.11	20,998	91.54	5.26 (4.80, 5.73)	1 (1, 271)
**Opioid prescription indication**^**f**^						
Hospice/end of life care	491	10.32	2,555	11.14	5.20 (4.80, 5.61)	3 (1, 31)
Cancer	2,193	46.11	14,260	62.17	6.50 (5.86, 7.14)	2 (1, 244)
Surgery pain	915	19.24	1,098	4.79	1.20 (1.16, 1.24)	1 (1, 10)
Acute pain	117	2.46	916	3.99	7.83 (4.13, 11.53)	2 (1, 169)
Chronic pain	153	3.22	1,340	5.84	8.76 (5.58, 11.94)	2 (1, 166)
Unknown	887	18.65	2,769	12.07	3.12 (2.45, 3.79)	1 (1, 186)

^a^Some members received opioid at different ages and could be counted in different age groups

^b^^‘^Other’ includes Asian (51 members), American Indian or Alaskan Native (15 members), Native Hawaiian/Pacific Islander (2 members), multi-racial (31 members), other (12 members)

^c^MME: morphine milligram equivalent

^d^ ≥ 90 days of supply in a 12-month period

^e^Not including members’ first opioid prescription claims paid by the Program as their previous history of opioid prescriptions was unknown

^f^Based on algorithms shown in Fig. 1

^g^Among members who received any opioid prescriptions, this column provides the mean and median number of opioid prescriptions that were dispensed to each member

### Trends of opioid prescriptions dispensed among the Program population during 2013–2021 (Fig. 2)

**Fig. 2:**
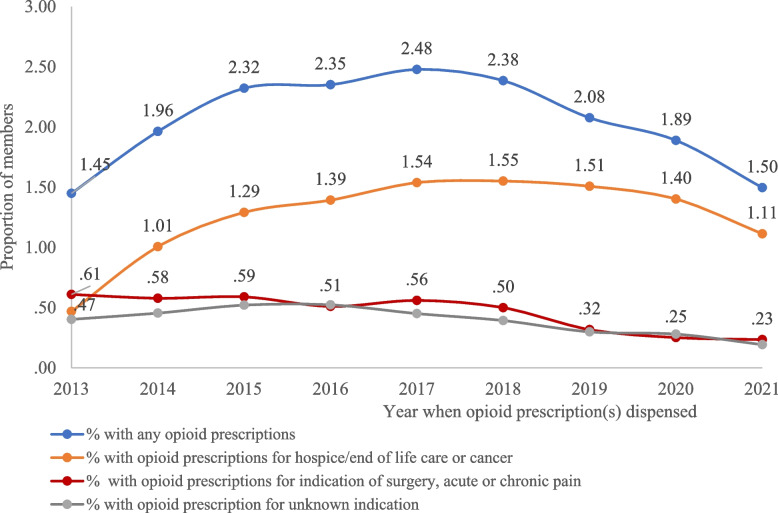
Proportion of members with diagnostic or treatment claims who had opioid prescription claims during 2013–2021

A total of 70,721 members had diagnostic/treatment-related claims paid by the Program. Among these members, there was an increasing trend of the proportion of members with any opioid prescription, from 1.5% in 2013 to 2.5% in 2017 (*p* = 0.024), then it decreased to 1.5% in 2021 (*p* = 0.003). A similar trend was observed in the proportion of members with outpatient opioid prescriptions for hospice/end of life care or cancer, with 0.5% in 2013, increasing to 1.6% in 2018 (*p* = 0.010), then decreased to 1.1% in 2021 (*p* = 0.070). The proportion of members with opioid prescription for surgery, acute or chronic pain decreased from 0.6% in 2013 to 0.23% in 2021 (*p* = 0.0005).

### PQA measures for opioid prescriptions during 2013–2021

**Fig. 3:**
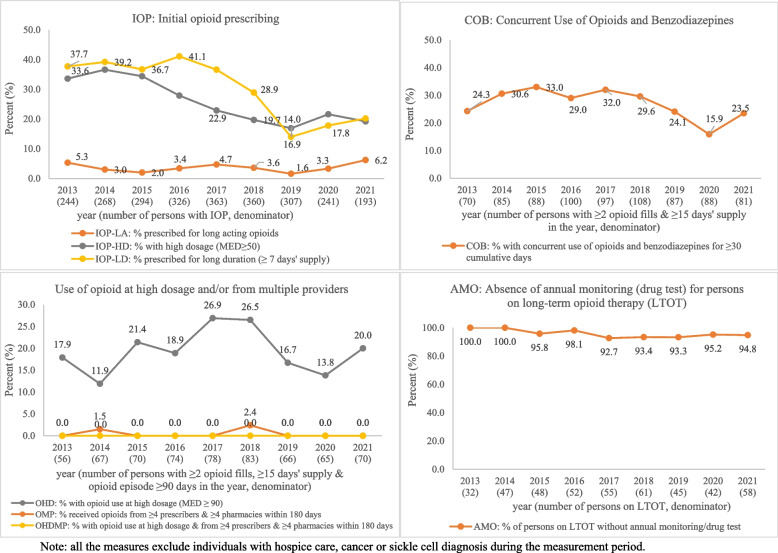
PQA measures on opioid prescriptions by year from 2013 to 2021 paid by the WTC Health Program

Figure 3 shows the trends of PQA measures for opioid prescriptions by year. Among the three measures related to initial opioid prescribing (IOP, new opioid prescription after ≥ 90 days without opioid use): IOP-LD, the proportion of individuals who had IOP for long duration (≥ 7 days’ supply), was around 37%-41% from 2013 to 2016, then decreased to 14% in 2019 and increased to 19% in 2021; IOP-HD, the proportion of individuals who had IOP at high dosage (≥ 50 MME/day), had been decreasing from 37% in 2014 to 17% in 2019, then increased to 20% in 2021; IOP-LA, the proportion of individuals who had IOP for long-acting opioids, had decreased from 5% to 2% in 2019 and then increased to 6% in 2021.

Three measures are related to use of opioid at high dose or from multiple providers (≥ 4 prescribers and ≥ 4 pharmacies). The population of evaluation interest is defined as persons with ≥ 2 opioid prescriptions dispensed, ≥ 15 days' supply, and opioid episode (from the first date of supply of the first opioid prescription dispensed to the last date of supply of the last opioid prescription dispensed in a measurement year) ≥ 90 days in the year and ranged from 56 individuals in 2013 to 83 in 2018. OHD, the proportion who had opioid use at high dosage (≥ 90 MME/day), increased from 18% in 2013 to 27% in 2017, then decreased to 14% in 2020, and increased to 20% in 2021. OMP, the proportion who received opioid from multiple providers within 180 days were zero in all years, except 1.5% in 2014 and 2.4% in 2018. OHDMP, the proportion with opioid use at high dosage and from multiple providers, were zero in all years.

Measure COB (concurrent use of opioids and benzodiazepines), percent of individuals with ≥ 2 opioid prescription dispenses and ≥ 15 days' supply who had COB for ≥ 30 cumulative days in the year, was approximately 30% from 2014 to 2018, then decreased to 16% in 2020 and increased to 24% in 2021. Measure AMO, the proportion of persons on long-term opioid therapy (LTOT, with ≥ 90 days’ cumulative supply in the year) who had no annual monitoring (drug test), was close to 100% in all years, indicating that drug testing among persons on LTOT was rare.

## Discussion

A total of 3.7% of the Program members received Program-paid outpatient opioid prescriptions from 2013 to 2021, with an annual prescription rate from 0.6% to 1.1%. This is much lower than the reported annual United States population opioid prescription rate of about 20% each year from 2014 to 2016 [20], 17% in 2017 [21] and 15% in 2018 [22]. Also, there were 21 opioid prescriptions dispensed per 100 Program members during the nine-year period, much lower than the reported annual rate of 78.1 in 2013 and 43.3 in 2020 per 100 persons in the United States [23]. These results were not unexpected as the WTC Health Program is a limited health care program that covers only conditions associated with the 9/11 terrorist attack. As such, this analysis did not include opioid prescriptions for non-WTC related conditions or paid by other insurance or self-pay, and the actual overall opioid prescription rate among Program members, when all health conditions are considered, may be higher than the reported rate in this study.

Members with hospice care or surgery procedures had the highest opioid prescription rates, followed by members with cancer, and acute or chronic pain claims. The majority of the opioid prescriptions were dispensed for cancer, hospice, or end-of-life care. This is not surprising as opioids are widely used for treatment of severe pain from cancer or those in hospice care, and nearly 20% of the Program population had a cancer certification. This study showed that members with PTSD but no cancer certifications had a higher prevalence of opioid prescriptions, compared to those with other non-cancer WTC Health Program conditions. This is consistent with the findings of a previous study [24], which used self-reported data to examine the association between PTSD and the risk of prescription opioid use and misuse among the World Trade Center Health Registry enrollees, who lived, worked or went to school in the area of the WTC disaster, or were involved in rescue and recovery efforts. That study found that individuals with past or current PTSD had a greater risk of opioid-related outcomes (i.e., prescription opioid use and misuse) compared to those who never had PTSD [24].

Among those with Program-paid diagnostic or treatment claims, opioid prescribing rates increased monotonically from 2013 to 2017, then decreased monotonically over the subsequent years. This trend was different from the national trend, which decreased from 21% in 2014 to 15% in 2018 [20–22], probably because the increase in our study was mostly driven by prescriptions for hospice/end-of-life care or cancer in an aging population exposed to 9/11 attacks, which increased from 2013 to 2018. The decrease since 2019 in our study was largely driven by the decreasing trend of opioid prescriptions for surgery, acute or chronic pain, and also the slow decrease for hospice/end-of-life care or cancer since 2019. This coincided with the various measures implemented by the Program (as listed in the background section) to improve opioid prescribing among its members, starting in September 2018, and after publication of the 2016 CDC Guideline [5]. Although overall opioid prescriptions were decreasing before the release of the 2016 CDC Guideline, the timing of this release was associated with an acceleration in decreases of overall prescribing and high-risk prescribing (e.g., high-dosage opioid prescribing and concurrent prescribing of opioid pain medication and benzodiazepines) [25–27]. Starting in March 2020, health care delivery in the United States was disrupted by the Coronavirus disease 2019 (COVID-19) pandemic. Studies have shown that the pandemic led to unchanged or decreased opioid prescribing [28–30]. Although our study was not intended to assess the impact of the pandemic on opioid prescribing paid by the Program, a clear impact was not observed in our study. A decreasing trend in opioid prescribing began in 2019 (pre-pandemic) and continued to 2021, and the reasons why the trend kept going down during the pandemic might involve the measures implemented in 2018 and/or pandemic impacts.

The PQA opioid measures were developed so that claims data could be used to evaluate opioid prescription safety using the 2016 CDC Guideline recommendations, and excluded individuals with hospice care, cancer, or sickle cell diseases. The 2016 CDC Guideline recommended that when starting opioid therapy, clinicians should prescribe immediate-release opioids, the lowest effective dosage, and no more than the quantity needed [5]. In this study, among members with IOP, less than 6.5% started with long-acting opioids (PQA measure IOP-LA) in each measurement year from 2013–2021, which is consistent with the CDC recommendation. Though the proportion of members with IOP who were prescribed for long duration (≥ 7 days, PQA measure IOP-LD) and the proportion started with high dosage opioids (≥ 50 MME/day, measure IOP-HD) were both relatively high, especially in 2013–2016 (between 28% -41%), both have decreased since 2017.

The 2016 CDC Guideline also recommended avoiding increasing the dosage to ≥ 90 MME/day without justification and avoiding COB prescribing whenever possible [5]. CMS also provided estimates of a few PQA opioid measures related to these recommendations, using claims data from Medicare Advantage Prescription Drug Contracting (MAPD) and Prescription Drug Plan (PDP) in 2019 and 2020 [31]. For the PQA measure OHD, use of high-dose opioids in persons without cancer, our study reported 16.7% in 2019 and 13.8% in 2020. These values are higher than the estimate reported by CMS, which was about 7% on average for both MAPD and PDP in 2019 and 2020 [31]. For concurrent use of opioids and benzodiazepines (PQA COB), the estimate in our study was higher than the CMS estimate in 2019 (24% vs 17%) but was similar to CMS in 2020 (16% vs 17%). For the PQA measures OMP, use of opioids from multiple providers in persons without cancer, and OHDMP, use of opioids at high dosage and from multiple providers, estimates in this study for each year from 2013 and 2021 were all close to zero, indicating good practice with respect to controlling the overdose risk factor from using opioids from multiple providers.

The Program semiannually reviews concurrent prescriptions of high dose opioids (> 90 MMEs/day) and benzodiazepines. When reviewing the medical records of 31 members from 2017 and 2018 with opioid > 90 MMEs/day or with benzodiazepine concurrent use for non-cancer treatment, the Program found that such use was medically necessary in 61% of instances. Although opioid use by some non-cancer members appears inconsistent with CDC 2016 recommendations based on claims data, the related benefits might outweigh the risks.

The 2016 CDC Guideline recommended urine drug testing (i.e., toxicology testing) before starting opioid therapy and consider testing at least annually for persons who were prescribed opioids for chronic pain to assess for the prescribed opioid as well as for other controlled prescription drugs and illicit drugs. The PQA measure AMO, annual monitoring for persons on long-term opioid therapy, in this study showed that annual drug monitoring had been rarely conducted during 2013–2021. It is possible that drug tests were conducted for the members but not paid by the Program and thus not captured by this analysis. Furthermore, the updated 2022 CDC Guideline recommended that clinicians consider the benefits and risks of toxicology testing to assess for prescribed medications as well as other prescribed and nonprescribed controlled substances when prescribing opioids for subacute or chronic pain [6].

There are several limitations with this study. First, electronic health records were not available for this analysis, making it challenging to identify the actual medical conditions related to opioid prescriptions. As a result, this study developed algorithms to estimate opioid prescription indicators, and 12% of opioid prescriptions during 2013–2022 had an unknown indicator. Second, only claims paid by the WTC Health Program were included. As such, data on the scope of opioid use and related services, such as annual urine drug tests, may be incomplete since claims paid by other insurance providers or by self-pay were not available. In addition, opioid prescriptions for conditions unrelated to WTC exposure (and thus not covered by the Program) were also not available. Third, chronic pain- and acute pain-related ICD-10-CM codes were abstracted from Mikosz et al. [17], and if there have been any subsequent updates to those pain-related ICD codes, such updates are not included in our analyses. In addition, diagnosis/treatment claims with pain-related ICD codes do not always indicate a pain diagnosis, as the ICD code might be for an exclusion diagnosis. Finally, since our study focused on the WTC Health Program population, the findings may not be generalizable to other populations. Nonetheless, given this population’s high prevalence of cancer, mental health conditions, and co-morbidities, this is an important group to study. In addition, other health programs may gain insights from the Program’s experience in managing opioid prescribing.

## Conclusions

Program-paid opioid prescription rates for all members and for those with non-cancer pains were low, and opioid prescriptions paid by the Program were primarily dispensed to members with cancer. Based on the trends of opioid prescription rates and practice, Program efforts to improve opioid prescribing coincided with improvements in outcomes. Nevertheless, potential areas for improvement were identified and continued surveillance is needed, such as urine drug tests during LTOT and prescribing opioids at high dose or concurrently with benzodiazepines to members without cancer.

## Data Availability

The datasets analyzed during the current study are not publicly available due to federal privacy restrictions, but deidentified data may be available from the World Trade Center Health Program through a valid and reasonable request. Please contact the corresponding author for requesting access to related data.

## Abbreviations

MED: Morphine milligram equivalents per day
NIOSH: National Institute for Occupational Safety and Health
CDC: Centers for Disease Control and Prevention
WTC: World Trade Center
MME: Morphine milligram equivalents
PQA: Pharmacy Quality Alliance
CPT: Current Procedural Terminology
ICD-CM: Classification of Disease, Clinical Modification
NDC: National Drug Codes
PTSD: Post-traumatic stress disorder
SUD: Substance use disorder
OSA: Obstructive sleep apnea
IOP: Initial opioid prescribing
IOP-LD: Initial opioid prescribing for long duration
IOP-LA: Initial opioid prescribing for long-acting opioids
IOP-HD: Initial opioid prescribing at high dose
OHD: Opioid use at high dosage
OMP: Opioid from multiple providers
OHDMP: Opioid use at high dosage and from multiple providers
COB: Concurrent use of opioids and benzodiazepines
AMO: Annual Monitoring for Persons on Long-Term Opioid Therapy
LTOT: Long-term opioid therapy
