# Correction: Opioid prescriptions among the World Trade Center Health Program population

**DOI:** 10.1186/s12913-024-11027-7

**Published:** 2024-04-30

**Authors:** Ruiling Liu, Geoffrey M. Calvert, Kristi R. Anderson, Helen Malcolm, Lauren Cimineri, Hannah Dupont, Marisol Martinez

**Affiliations:** World Trade Center Health Program, National Institute for Occupational Safety and Health (NIOSH), Centers for Disease Control and Prevention (CDC), Atlanta, GA 30329 USA

**Correction to:** ***BMC Health Services Research*** **(2023) 23: 1323.**

10.1186/s12913-023-10233-z

In the Authors’ Contributions section of this article, the authors’ credentials for some of the authors were incorrect. They were incorrectly given as “Drs. Anderson, Malcolm, Cimineri, Dupont and Martinez” and should have been “Dr. Anderson, Ms. Malcolm, Dr. Cimineri, Ms. Dupont and Dr. Martinez”.

The original article has been updated.

